# Completion Thyroidectomy Trends and Rates: A Systematic Review and Meta‐Analysis

**DOI:** 10.1111/coa.14262

**Published:** 2024-11-26

**Authors:** Daniel Soibelman, Ohad Ronen

**Affiliations:** ^1^ Azrieli Faculty of Medicine Bar‐Ilan University Safed Israel; ^2^ Department of Otolaryngology – Head and Neck Surgery Galilee Medical Center Nahariya Israel

**Keywords:** guidelines, meta‐analysis, systematic review, thyroid carcinoma, thyroidectomy

## Abstract

**Background:**

In January 2016, the American Thyroid Association (ATA) published an update to the guidelines concerning the management of adult patients with thyroid nodules and well‐differentiated thyroid cancers. One of the revised recommendations states that lobectomy is a reasonable surgical approach for low‐risk patients. This systematic review compares the rates of completion thyroidectomy surgeries before and after the publication of the recent ATA guidelines.

**Methods:**

A systematic review was conducted according to the PRISMA guidelines of the preferred reporting items for systematic reviews and meta‐analyses. PubMed and Embase databases were searched to find articles which demonstrate the rates of completion thyroidectomy surgeries in the last 6 years, before and after the recent ATA guidelines publication. Overall, 8744 titles and abstracts were screened, and 964 articles were fully assessed for eligibility. Eventually, 40 studies were included for data extraction. More than 48 000 patients with thyroid malignancy were included in the review, and were divided into three time periods according to the publication date of 2015 ATA guidelines.

**Results:**

We found that the rate of completion thyroidectomy was 51.8% before 2016 and 43.1% after the 2015 ATA guidelines publication. We observed a 17% reduction of early completion thyroidectomy surgeries since the 2015 ATA guidelines publication relative to previous periods, among patients with malignant pathology.

**Conclusions:**

Apparently, more centres worldwide implemented the new guidelines and prefer a conservative surgical approach as compared to the pre‐ATA 2015 era.

**Summary**

- We investigated whether there was a change in the completion thyroidectomy surgeries rate before and after the publication of the 2015 ATA guidelines concerning the management of adult patients with thyroid nodules and well‐differentiated thyroid cancers.
- We found that the rate of completion thyroidectomy was 51.8% before 2016 and 43.1% after the 2015 ATA guidelines publication. We observed a 17% reduction of early completion thyroidectomy surgeries since the 2015 ATA guidelines publication relative to previous periods among patients with malignant pathology.
- Apparently, more centers worldwide implemented the new guidelines and prefer conservative surgical approach compared to the pre‐ATA 2015 era.

**Abbreviations**
ATA: American Thyroid Association
WDTC: well‐differentiated thyroid cancers

## 1 Introduction

Thyroid cancer is the most abundant endocrine malignancy worldwide. In the United States alone, it is represents 1%–3% of all diagnosed malignancies among males and females, respectively [1]. Thyroidectomy is the main treatment for a large degree of thyroid gland malignancies, including some of the benign tumours. Common complications include‐ superior laryngeal nerve palsy, hypoparathyroidism and haemorrhage [2]. Thyroidectomy surgery evolved throughout the years in order to reduce the complications and side effects. Today it is categorised into: total thyroidectomy, hemithyroidectomy, also known as lobectomy, and near‐total thyroidectomy [3, 4]. In certain circumstances, after lobectomy surgery, early completion thyroidectomy procedure should be performed [5, 6].

Well‐differentiated thyroid cancers (WDTC) represent more than 90% of thyroid malignancies, including papillary and follicular thyroid carcinoma. Few aspects of thyroid cancer management, including early completion thyroidectomy are still under debate in the last decade as represented in the American Thyroid Association (ATA) guidelines concerning the management of adult patients with thyroid nodules and well differentiated thyroid cancer [7, 8].

The last ATA guidelines were published at the beginning of 2016 (ATA 2015 guidelines). Recommendation 35B states that either thyroidectomy or lobectomy is a reasonable surgical approach for patients with DTC measuring 1–4 cm without evidence of extrathyroidal extension or clinically apparent lymph node disease, as a majority of studies did not demonstrate negative impaction of lobectomy as compared to thyroidectomy regarding overall survival and disease‐free survival [9]. Therefore, this recommendation allows considerable latitude in choosing the extent of surgery.

According to the ATA 2015 guidelines, if lobectomy was performed, completion thyroidectomy should be offered to patients for whom a bilateral thyroidectomy would have been recommended had the diagnosis been available before the initial surgery. Another recommendation, based on low‐quality evidence, states that completion thyroidectomy may be necessary when the diagnosis of malignancy is made following lobectomy for an indeterminate or nondiagnostic biopsy. Also, completion of thyroidectomy may be required to provide complete resection of multicentric disease and allow efficient RAI therapy [8]. Notably, Recommendation B8 in the previously published ATA 2009 guidelines states that for patients with thyroid cancer > 1 cm, the initial surgical procedure should be a near total or total thyroidectomy [10].

Following the new ATA guidelines and the controversies mentioned above, we decided to conduct a systematic review in order to examine the trends and rates of completion thyroidectomy in clinical practice following publication of the ATA 2015 guidelines. According to the significant changes between the ATA 2015 and ATA 2009 guidelines regarding the extent of thyroidectomy surgery, we hypothesize that a reduced rate of early completion thyroidectomy surgeries would be observed since 2016, relative to studies performed before the new guidelines publication.

The data provided from the review may serve as another tool for surgeons in order to make an informed decision with the patient regarding the extent of thyroidectomy operation.

## 2 Materials and Methods

### 2.1 Ethical Approval

Not applicable as this article is based on previously conducted studies and does not contain any new studies with human participants or animals performed by any of the authors.

### 2.2 Search Strategy and Databases

The systematic review was conducted according to the guidelines of the preferred reporting items for systematic reviews and meta‐analyses [11, 12].

The electronic search of the literature was conducted in PubMed and EMBASE. We searched for articles that were published from January 2014 through October 2020. This timeframe was chosen because the ATA 2015 guidelines, which were published in January 2016, were the point of intervention, therefore we screened the literature in order to explore the trends of completion thyroidectomy before and after this event. We searched for observational and interventional studies; therefore, case reports, letters and surveys were excluded. We used “Thyroidectomy” as a key word and searched for studies in English with participants above the age of 18. The references of each article obtained were checked for additional relevant studies.

### 2.3 Data Selection and Extraction

Study selection was conducted by independent two reviewers (O.R. and D.S.) who screened articles for eligibility, based on the research question and on the inclusion and exclusion criteria. Studies were chosen for data extraction after consensus was reached between the reviewers. Data for the studies were extracted using standardised data forms. The data included—author, publication year and country, the years in which surgeries were performed, age and gender of the population, amount of total and partial thyroidectomy surgeries, number of completion thyroidectomy procedures, malignancy percentage, Bethesda classification score, frozen section, and final pathology results.

### 2.4 Inclusion Criteria

Completion/conversion of partial to total thyroidectomy surgeries which were performed during the first 6 months after the initial surgery; whether it was possible to extract the amount of the completion surgeries out of the partial thyroidectomy population; the patients included in the study did not undergo previous thyroid operations. Data regarding the total malignancy percentage of the thyroid gland was obtainable in order to compare properly between populations.

### 2.5 Exclusion Criteria

Studies involving surgeries for recurrent thyroid malignancy were excluded; completion thyroidectomy surgeries performed after a follow‐up of more than 6 months following partial thyroidectomy were excluded. It should be noted that study designs which retrospectively included only patients who had malignant final pathology and therefore would present higher completion percentage rates—were excluded.

### 2.6 Quality Appraisal

Newcastle‐Ottawa scale for cohort studies was used for articles assessment [13]. Selection of participants (maximum 4 points), comparability (maximum 2 points) and assessment of outcome (maximum 3 points) was evaluated for each study. Scores of 7–9, 4–6 and 0–3 indicate good, moderate and poor study quality, respectively [14].

### 2.7 Data Synthesis

The Freeman‐Tukey transformation (arcsine square root transformation) was used to calculate the weighted summary of the completion thyroidectomy proportions across studies. Due to large study heterogeneity, as demonstrated in the Results section below and Cochran's Q measure, the random effects proportion was used. The chi‐square test was used to compare these proportions, before and after 2015. The Medcalc application, V20.009 was used for all calculations and two‐sided *p*‐values less than 0.05 were considered statistically significant.

## 3 Results

### 3.1 Study Selection

**FIGURE 1:**
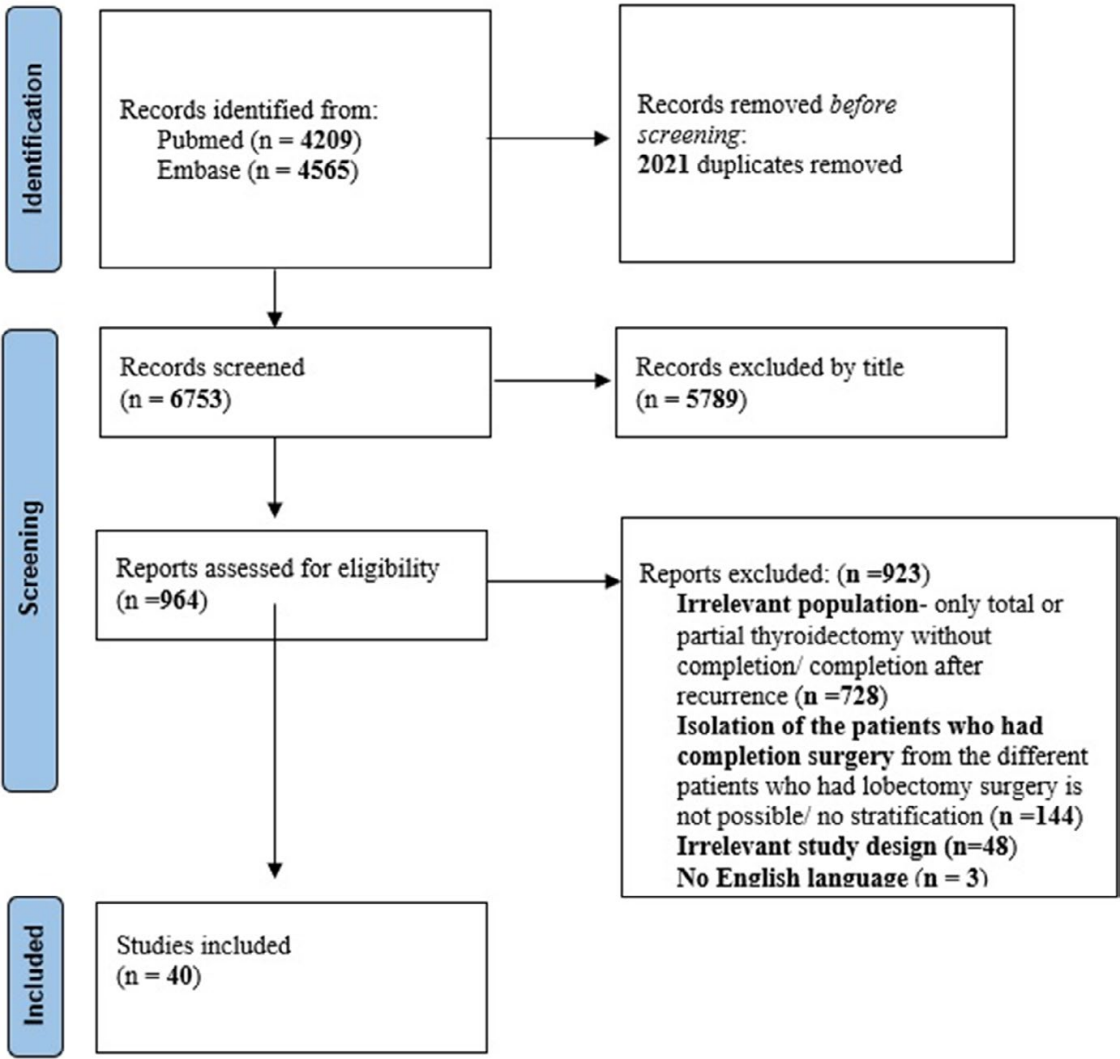
PRISMA flow diagram of search strategy.

The search strategy identified a total of 8774 studies (Figure 1): 4209 from PubMed and 4565 from Embase. The authors screened the titles and the abstracts and found 964 articles (out of 6753 and 8744 studies, respectively after duplicates were removed) that were fully assessed for eligibility. Eventually, 40 articles were selected for data extraction (Tables 1, 2, 3). The main reasons articles were excluded were: (1) The study population did not meet our inclusion criteria, mostly because the patients were not treated by completion thyroidectomy (either single‐stage total thyroidectomy or hemithyroidectomy without completion), or the completion operation was performed because of disease recurrence several years after the first procedure. (2) The articles described treatment with completion thyroidectomy, but the specific number of the procedures and the patients could not be separated from the total/partial surgeries. (3) Articles in the form of letters, surveys and economic models were excluded.

**TABLE 1:** Publications of only malignancy cases—before ATA 2015 guidelines.

Author	Year of publication	Country	Number of patients	Median age	SD	Mean age	Female percentage	First year of surgeries	Last year of surgeries	Total thyroidectomy	Hemithyroidectomy	Completion	Completion percentage
Hirshoren N [17]^a^	2018	Israel	118		14	45	78	2013	2014	72	46	34	73.9
Dobrinja C [33]^b^	2017	Italy	86			54	76.7	2001	2016	86			
Dobrinja C [33]^b^	2017	Italy	19			56	73.6	2001	2016		19	8	42.1
Price AK [19]	2017	United States	125		14.6	50.1	85.6	2008	2016	91	34	10	29.4
Kiernan CM [34]	2017	United States	17	51				2009	2016	13	4	3	75
Chereau N [35]^c^	2016	France	1840			52	82	1978	2014	1451	389	217	55.7
Chereau N [35]^d^	2016	France	678			46	80	1978	2014	507	171	98	57.3
Adkisson CD [36]	2014	United States	359			53	77	2011	2011	260	99	81	81.8
Hall SF [37]	2014	Canada	12 959			47.1	80	1999	2008	8095	4864	2636	54.1
Sugino K [38]	2014	Japan	324	45			78	1989	2010		324	101	31.1
Park YM [39]	2014	Korea	268			51.6	90	2013	2013	254	14	5	35.7
Sawant R [40]	2019	United Kingdom	361	45			81	2009	2015	156	205	161	78.5
Kluijfhout WP [16]	2017	United States	394		14	45	79.2	2000	2010	310	84	74	88
Kuo LE [41]^e^	2020	United States	63		17.4	47	74.6	2014	2015		63	41	65
Ullmann TM [42]^f^	2019	United States	23 751		14	54	76.4	2009	2015	17 675	4100	1976	48.1
Zambeli‐Ljepović A [18]	2019	United States	3214		5.5	72.8	77.6	1996	2011	2103	1111	180	16.2
Lee JC [43]^g^	2019	Australia	333		15	49.6	79	2007	2010	226	107	14	13
Lee JC [43]^h^	2019	Australia	342		14.5	51.8	75	2011	2013	253	89	12	13.4
Al‐Qurayshi Z [44]^i^	2020	United States	138 773		14.6	48.6	79.3	2004	2014	106 755	32 018	19 951	62.3
Al‐Qurayshi Z [44]^j^	2020	United States	13 614		14.6	48.6	59.1	2004	2014	9350	4264	3095	72.5

^a^ Pre ATA2015 guidelines in comparison to post ATA2015 cohort in the same study.

^b^ Hemithyroidectomy was no performed in this cohort.

^c^ T1a histopathology patients cohort, in comparison to T1b patients in the first cohort.

^d^ T1b histopathology patients cohort, in comparison to T1a patients in the first cohort.

^e^ Pre ATA2015 guidelines in comparison to post ATA2015 cohort in the same study.

^f^ Pre ATA2015 guidelines in comparison to post ATA2015 cohort in the same study.

^g^ Pre ATA2009 guidelines in comparison to post ATA2009 cohort in the same study.

^h^ Post ATA2009 guidelines in comparison to pre ATA2009 cohort in the same study.

^i^ Tumour size below 4 cm in comparison to above 4 cm cohort in the same study.

^j^ Tumour size above 4 cm in comparison to below 4 cm cohort in the same study.

**TABLE 2:** Publications of only malignancy cases—time period including the 2015 ATA guidelines.

Author	Publication year	Country	Number of patients	Median age	Mean age	Female (%)	First year of surgeries	Last year of surgeries	Total thyroidectomy	Hemithyroidectomy	Completion	Completion percentage
Owens PW [45]	2018	Ireland, United Kingdom	178	43.5		73	2014	2017	67	111	105	94.5
Moore EC [46]^a^	2020	United States	298		54.5	80	2015	2018	205	93	18	19.3
Moore EC [46]^b^	2020	United States	170		54.5	80	2015	2019	108	62	26	41.9
DiMarco AN [47]	2019	Australia	275	51		82.9	2013	2017		275	117	42.5

^a^ Indeterminate Fine Needle Aspiration Cytology nodules group.

^b^ High risk FNAC nodules group.

**TABLE 3:** Publications with only malignancy cases—after the 2015 ATA guidelines.

Author	Year of publication	Country	Number of patients	Median age	SD	Mean age	Female percentage	First year of surgeries	Last year of surgeries	Total thyroidectomy	Hemithyroidectomy	Completion	Completion percentage
Hirshoren N [17]^a^	2018	Israel	51		13.5	44	74	2016	2016	16	35	7	20
Kuo LE [41]^b^	2020	United States	100		16.7	48.2	73	2016	2018		100	43	43
Nayyar SS [48]	2020	India	228	40			62.5	2017	2019	133	95	68	71.55
Ullmann TM [42]^c^	2019	United States	11 577		15.2	50.3	75.9	2015	2017	8116	2542	919	36.1

^a^ Post ATA2015 guidelines in comparison to pre ATA2015 cohort in the same study.

^b^ Post ATA2015 guidelines in comparison to pre ATA2015 cohort in the same study.

^c^ Post ATA2015 guidelines in comparison to pre ATA2015 cohort in the same study.

**TABLE 4:** Malignant and benign tumours—before ATA 2015 guidelines.

Author	Year of publication	Country	Number of patients	Median age	SD	Mean age	Female percentage	First year of surgeries	Last year of surgeries	Total thyroidectomy	Hemithyroidectomy	Completion	Completion percentage
Angell TE [49]	2018	United States	325		14.4	50.6	77.2	2012	2016	83	182	36	19.7
Bollig CA [50]	2018	United States	65		14.9	49.6	81.5	2010	2015	0	65	17	26.1
Cakir Bekir [51]	2018	Turkey	47		13.3	40.3	85	2009	2015	0	47	11	23.4
Schneider DF [52]	2017	United States	639		14.7	52	78.5	1994	2015	263	376	59	15.6
Abu‐Ghanem S [53]	2016	Israel	47		14.5	51	72.3	2010	2015		47	23	48.9
Jooya A [54]	2016	Canada	96		12.2	50.7	82.3	2011	2015	60	36	3	8.3
Rago T [31]	2014	Italy	1520		13.2	46.1		2000	2010	882	638	68	10.6
Conzo G [55]	2014	Italy	472		13.3	45.5	69	2000	2008	154	318	51	16
Stewart R [56]^a^	2020	Australia	2582		41.3	55.1	80	2001	2015	1578	1004	110	10.9
Stewart R [56]^b^	2020	Australia	1239		26.5	52.1	80	2001	2016	385	854	145	16.9
Ajarma KY [57]	2020	Jordan	567		14.5	44.3	76.5	2013	2016	185	360	56	15.5
Sarfati‐Lebreton M [58]	2019	France	493		14.3	46.5	77.8	2010	2014		158	18	11.3
Montgomery J [30]	2016	United Kingdom	65		16.8	51	69	2010	2014	44	16	11	68.7
Cotton TM [59]^c^	2016	United States	65			45.9	72	2008	2014	0	65	6	9.2
Cotton TM [59]^d^	2016	United States	45			46.6	71	2008	2014	0	45	5	11.1
Cotton TM [59]^e^	2016	United States	25			45.6	72	2008	2014	0	25	4	16
Berg RW [29]	2016	United States	235	52			84	2009	2013		235	12	5.1
Vuong CD [60]	2020	United States	314					2007	2013		314	25	7.9

^a^ Definitive fine needle aspiration cytology—benign or malignant group.

^b^ Indeterminate fine needle aspiration cytology—indeterminate follicular cytology or suspicious for malignancy group.

^c^ FNA diagnosis of follicular lesion group.

^d^ FNA diagnosis of Bethesda 3 group.

^e^ FNA diagnosis of Bethesda 4 group.

**TABLE 5:** —Malignant and benign tumours—time period including before and after ATA 2015 guidelines.

Author	Year of publication	Country	Number of patients	SD	Mean age	Female percentage	First year of surgeries	Last year of surgeries	Total thyroidectomy	Hemithyroidectomy	Completion	Completion percentage
Mallick R [61]	2019	United States	236	14.1	55.6	83	2015	2017	81	155	8	5.1
Awad B [62]	2019	Saudi Arabia	111	15.5	42.6	87.4	2013	2017		111	53	47.7
Pasha HA [63]	2020	Pakistan	81	13.9	43	85.2	2015	2017	39	42	8	19
Carty SE [64]^a^	2020	United States	182	15.6	54	73	2014	2019	93	88	14	15.9
Carty SE [64]^b^	2020	United States	29	15.7	54	73	2014	2020	18	11	2	18.1

^a^ Molecular testing was performed prior to thyroidectomy.

^b^ Molecular testing was not performed prior to thyroidectomy.

### 3.2 Studies Description and Synthesis of Data

A detailed description of the studies is provided in Tables 2, 3, 4, 5, 6. Nineteen studies were conducted within the United States, three each in Australia, Italy and the United Kingdom, two each in Israel, Canada and France, one each in India, Turkey, Jordan, Saudi Arabia, Japan, Korea and Pakistan. Due to coherent study design differences, we divided the study population into two groups: Group 1 consisted of studies that included only patients who had a histologically proven thyroid malignancy; Group 2 consisted of studies that included patients that had both malignant and benign thyroid tumours. Due to insufficient data in these articles, it was impossible to match between the final pathology and completion surgery, therefore a separate group was created. Another subdivision within the study population was made according to the time period of the partial and completion thyroidectomy surgery relative to the publication date of the ATA guidelines: before 2016, after 2016 and one in which the surgeries were performed during a period of time that included 2016. In the malignant‐only group there were 48 005, 541, and 2772 patients before, including the year 2016 and after 2016 respectively. The malignant and benign tumours group consisted of 8841, 639 and 538 patients before, including the year 2016 and after 2016, respectively. Female gender was the majority in all the studies. The main outcome in each study is the proportion of the patients who underwent completion thyroidectomy out of the patients who initially were treated by partial lobectomy.

**TABLE 6:** Malignant and benign tumours—after 2015 ATA guidelines.

Author	Year of publication	Country	Number of patients	Median age	SD	Mean age	Female percentage	First year of surgeries	Last year of surgeries	Total thyroidectomy	Hemithyroidectomy	Completion	Completion percentage
Linhares SM [32]	2020	United States	538		13	49	85	2016	2019	412	126	12	9.5

Group 1—malignant only: As mentioned above, 48 005 patients were enrolled for the studies in which the surgeries were performed before the 2015 ATA guidelines publication. In a meta‐analysis, the proportion of the completion thyroidectomy patients out of the partial thyroidectomy in random effects model was 51.8% (95% CI, 44.89–58.77). Considerable between‐study heterogeneity was found (I^2^ = 99.14%, *p* < 0.0001, CI, 98.99 to 99.27). In the subgroup which consisted of patients who underwent surgeries in the time period including 2016, the proportion on completion thyroidectomy was 51.5% (95% CI, 19.27–83.17). Furthermore, considerable between‐study heterogeneity was found (I^2^ = 98.33%, *p* < 0.0001, CI, 97.29 to 98.96). In the subgroup which included patients who underwent surgeries after the 2015 ATA guidelines publication, the proportion of completion thyroidectomy out of partial thyroidectomy was 43.1% (95% CI, 26.46–60.45). Additionally, considerable between‐study heterogeneity was found (I^2^ = 94.4%, *p* < 0.0001, CI, 88.98 to 97.24). Chi square test was made to compare the proportion between the three time periods, and there was statistical evidence for a difference of reduced completion thyroidectomy rate shown between the group after the ATA 2015 guidelines as compared to the group prior these guidelines (*p* < 0.001).

Group 2—Malignant and benign tumours: overall, 10 018 patients were enrolled for the studies in the three time periods. In a meta‐analysis, in the subgroup which included surgeries before the ATA 2015 guidelines, the proportion of completion thyroidectomy out of partial thyroidectomy in random effects model was 13.5% (95% CI, 12.76 to 19.02). Considerable between‐study heterogeneity was found (I^2^ = 86.45%, *p* < 0.0001, CI, 80.02 to 90.81). In the subgroup which consisted of patients who underwent surgeries in the time period including the ATA 2015 guidelines, the proportion of completion thyroidectomy was 20.4% (95% CI, 5.99 to 40.55). In addition, considerable between‐study heterogeneity was found (I^2^ = 94.53%, *p* < 0.0001, 95% CI, 90.00 to 97.01). Only one study in which the surgeries were performed after the 2015 ATA guidelines was included. The study included 538 patients with a completion thyroidectomy rate of 9.5%. Chi‐square test was also made to compare the proportion between the three time periods, the rate reduction from 13.5% to 9.5% after the ATA 2015 guidelines was not statistically significant. A difference of 10.9% between the subgroup than included patients before and after 2016 and the after 2016 group was statistically significant.

**FIGURE 2:**
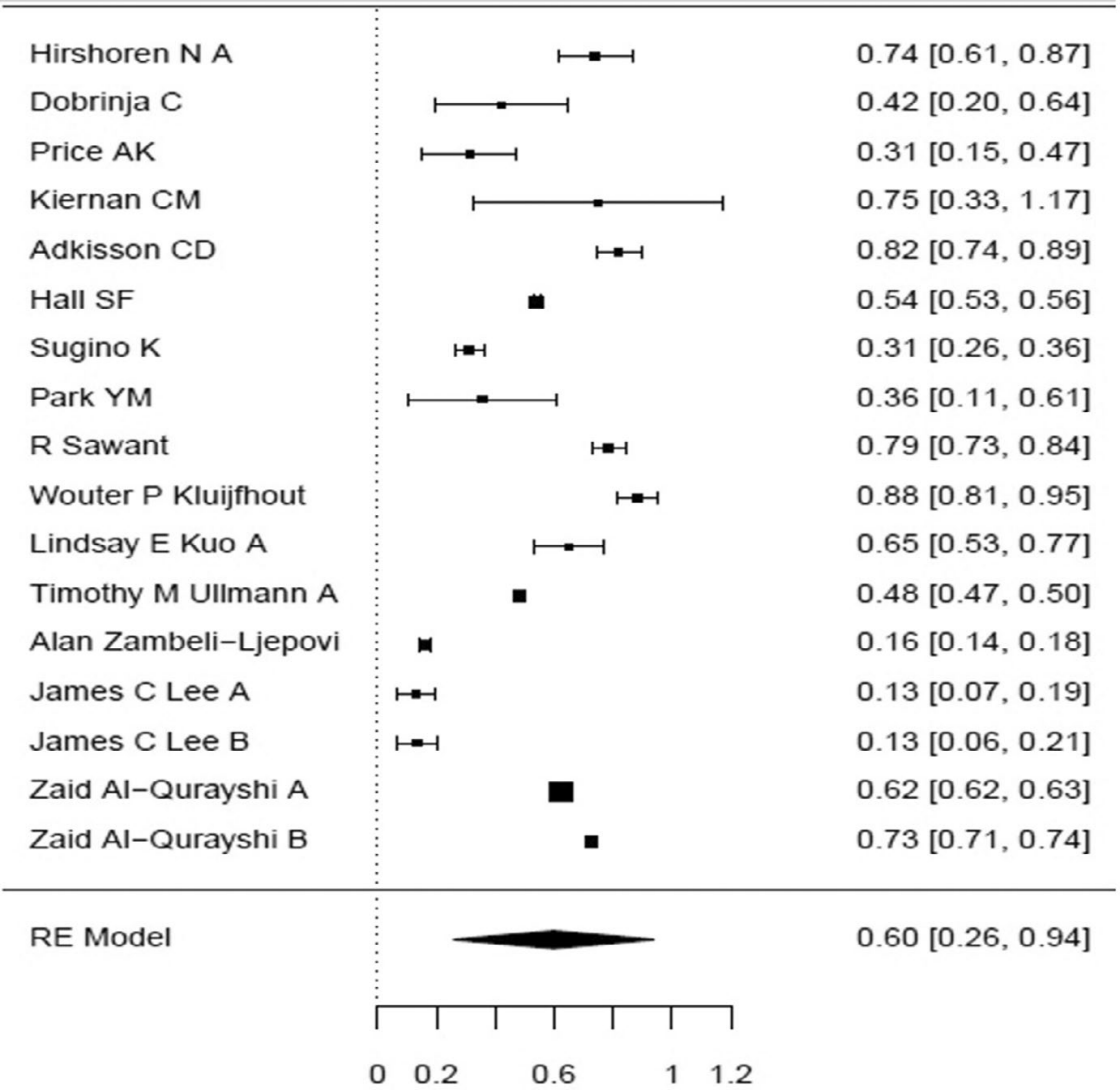
Forest plot showing the completion rate thyroidectomy of publications of malignant‐only cases—before 2016.

**FIGURE 3:**
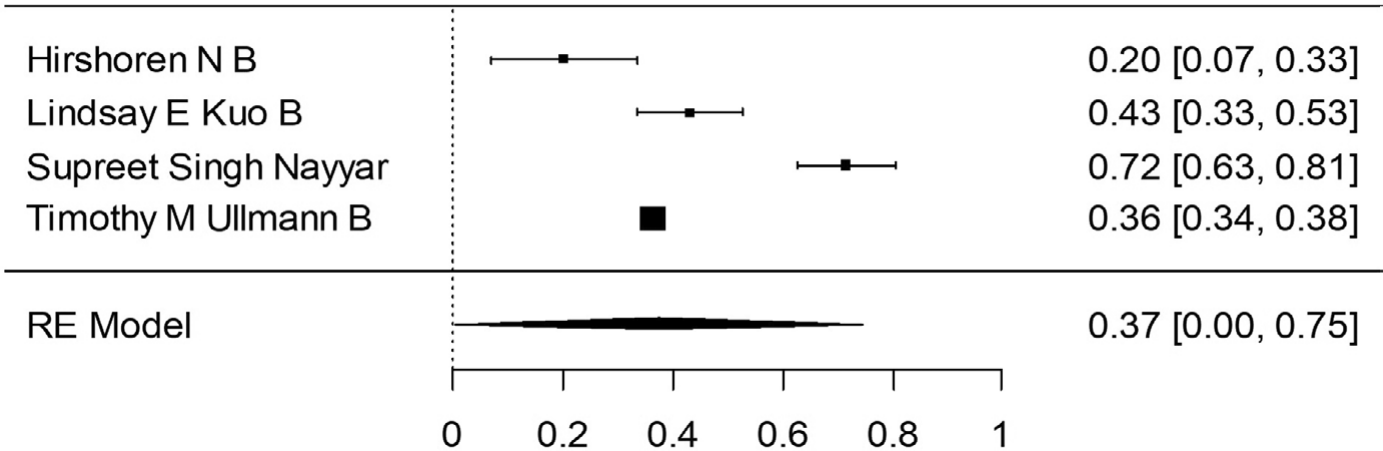
Forest plot showing the completion thyroidectomy rate of publications of malignant only cases—after 2016.

**FIGURE 4:**
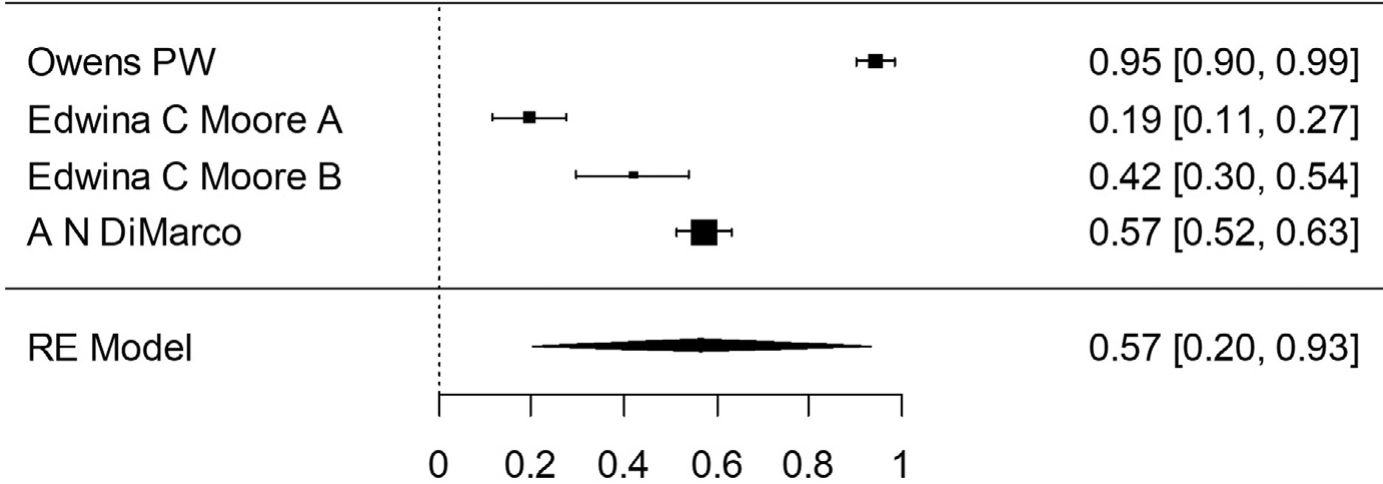
Forest plot showing the completion thyroidectomy rate of publications of malignant only cases—including 2016.

**FIGURE 5:**
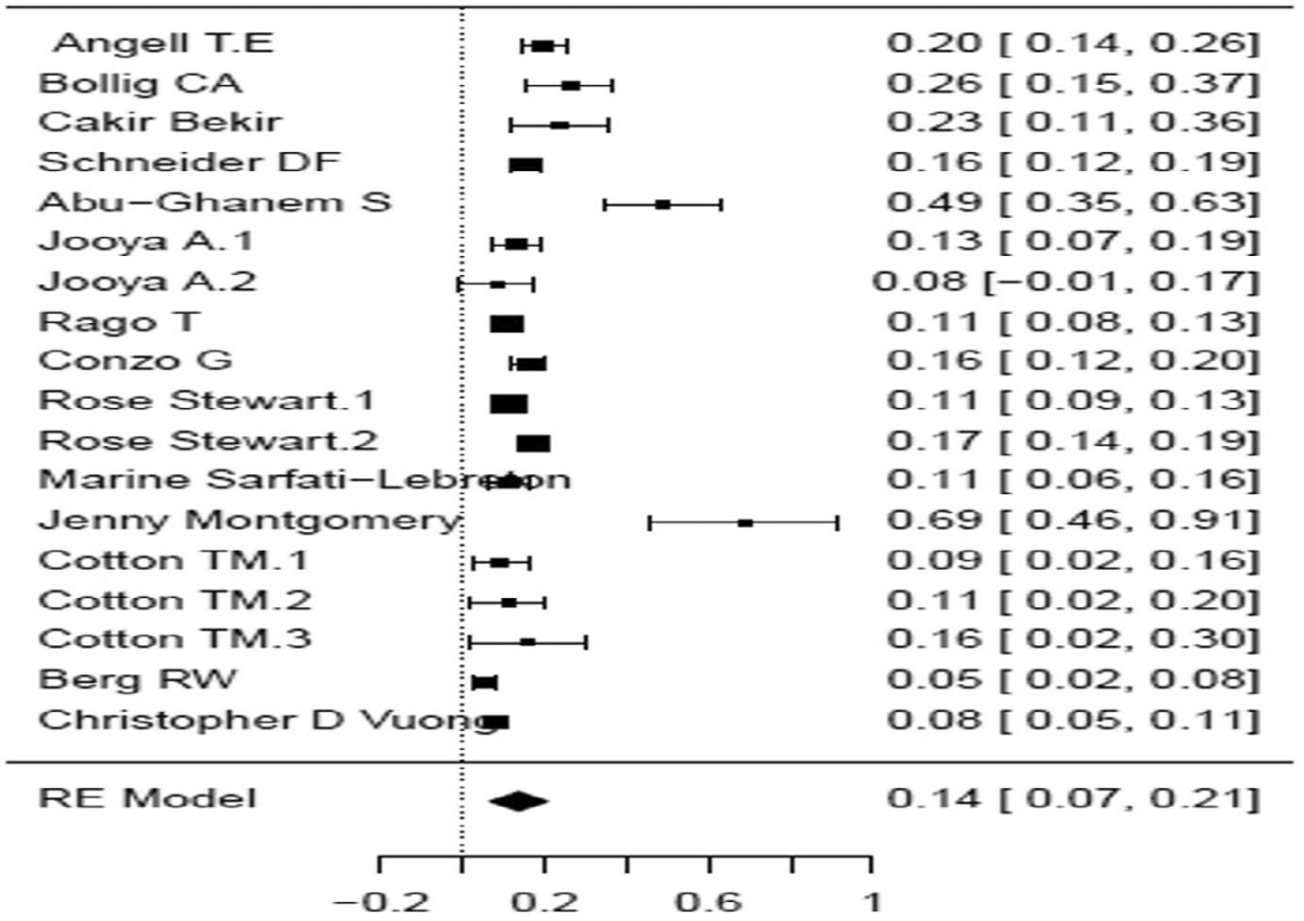
Forest plot showing the completion thyroidectomy rate of publications of malignant and benign cases—before 2016.

**FIGURE 6:**
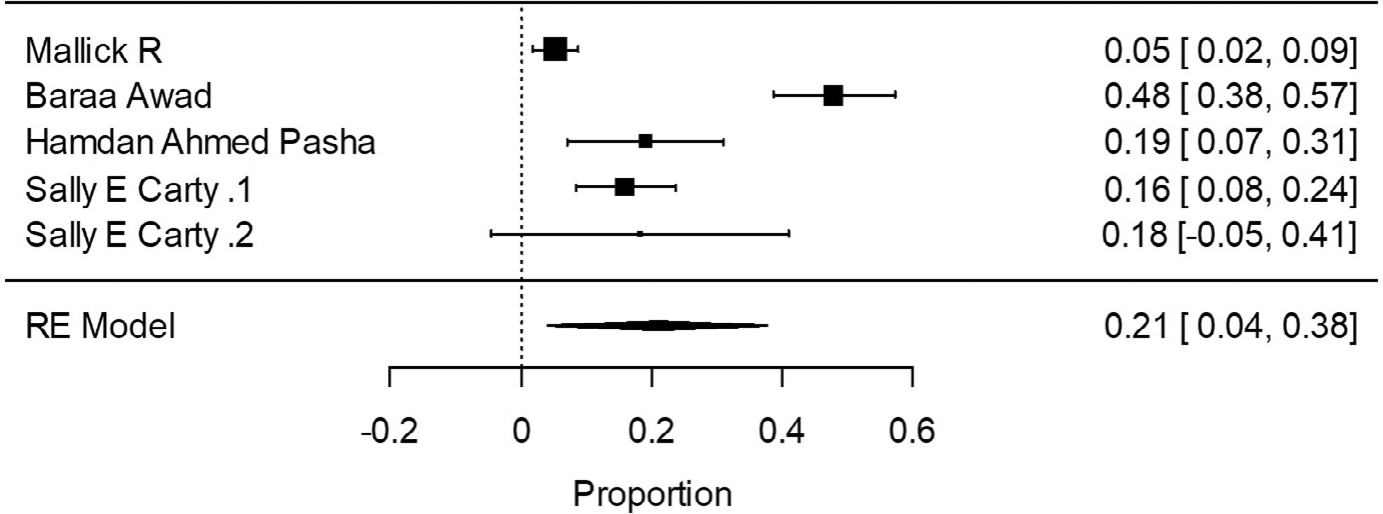
Forest plot showing the completion thyroidectomy rate of publications including malignant and benign cases—including 2016.

A forest plot was made to compare the data retrieved from the articles in the different groups and subgroups mentioned above (Figures 2, 3, 4, 5, 6).

### 3.3 Quality of Included Studies

One study was of high quality, 38 studies were of moderate quality and two studies were of low quality on the Newcastle–Ottawa scale (Table 7). Studies were mostly downgraded due to an absence of a control or comparison group.

**TABLE 7:** Quality of studies assessment with Newcastle–Ottawa scale.

Author	Publication year	Selection	Comparability	Outcome assessment	Total score
Hirshoren N [17]	2018	4	0	3	7
Dobrinja C [33]	2017	3	0	3	6
Price AK [19]	2017	2	0	3	5
Kiernan CM [34]	2017	2	0	2	4
Chereau N [35]	2016	2	0	3	5
Adkisson CD [36]	2014	2	0	3	5
Hall SF [37]	2014	2	0	1	3
Sugino K [38]	2014	3	0	3	6
Park YM [39]	2014	2	0	3	5
Sawant R [40]	2019	2	0	3	5
Kluijfhout WP [16]	2017	2	0	3	5
Kuo LE [41]	2020	3	0	3	6
Ullmann TM [42]	2019	2	0	2	4
Zambeli‐Ljepović A [18]	2019	2	0	2	4
Lee JC [43]	2019	2	0	1	3
Al‐Qurayshi Z [44]	2020	3	0	3	6
Owens PW [45]	2018	2	0	3	5
Moore EC [46]	2020	2	0	2	4
DiMarco AN [47]	2019	2	0	3	5
Nayyar SS [48]	2020	3	1	2	6
Angell TE [49]	2018	2	0	3	5
Bollig CA [50]	2018	2	0	2	4
Bekir C [51]	2018	2	0	2	4
Schneider DF [52]	2017	3	0	3	6
Abu‐Ghanem S [53]	2016	3	0	0	6
Jooya A [54]	2016	2	0	3	5
Rago T [31]	2014	3	0	3	6
Conzo G [55]	2014	3	0	3	6
Stewart R [56]	2020	3	0	3	6
Ajarma KY [57]	2020	2	0	3	5
Sarfati‐Lebreton M [58]	2019	3	0	0	6
Montgomery J [30]	2016	2	0	3	5
Cotton TM [59]	2016	3	0	3	6
Berg RW [29]	2016	2	0	3	5
Vuong CD [60]	2020	2	0	2	4
Mallick R [61]	2019	2	0	2	4
Awad B [62]	2019	3	0	3	6
Pasha HA [63]	2020	3	0	0	6
Carty SE [64]	2020	3	0	0	6
Linhares SM [32]	2020	3	0	3	6

## 4 Discussion

According to our study, since the publication of the ATA 2015 guidelines in January 2016, completion thyroidectomy rates were significantly reduced among many medical centres worldwide. In recent years, only 43.1% of patients with diagnosed thyroid malignancy were required to undergo second early operation, as compared to almost 52% of patients before 2016. Therefore, a 17% reduction in completion thyroidectomy surgery was observed. Out of 9 studies describing surgeries performed after January 2016, 7 (mostly in North American medical centres) show less than 43% of completion thyroidectomy, while only one study performed between 2017 and 2019 in India shows relatively high completion rates.

The Kluijfhout et al. study from 2016 analyzed 1000 patients who underwent thyroidectomy between 2000 and 2010 [15]. Their study tried to predict what would be the completion surgery rate of tumours between 1 and 4 cm if the ATA 2015 guidelines were available during those years. The study found that 43% of patients who underwent thyroidectomy for preoperatively low‐risk 1–4 cm WDTC would have needed a completion total thyroidectomy after pathological analysis of the initial lobe. Surprisingly, our study found precisely the same percentage of completion thyroidectomy surgeries actually performed after January 2016.

Although a 17% reduction in completion surgeries was observed between time periods, we would expect a larger scale of decline. The Kluijfhout et al. study from 2017 found that 88% of patients admitted for lobectomy between 2000 and 2010, required a completion operation [16]. Hirshoren et al. was the first study which was conducted to compare between completion thyroidectomy rates before and after ATA 2015 guidelines, and found that 73.9% of patients eligible for lobectomy underwent completion surgery before 2015 [17]. Our systematic review and meta‐analysis showed a relatively low completion rate of 52% before 2016. One of the explanations is based on the exclusion criteria of two large population studies: Zambeli‐Ljepović et al. study that consisted out of 1111 patients after tumours larger than 2 cm and cancers in situ were excluded, and Price et al. included only micro‐carcinoma patients [18, 19]. Another possible explanation is based on our review inclusion criteria. We included articles published in the past 5 years that were divided into before and after 2016. In many of the “before 2016” group, surgeries were performed a short time before the ATA 2015 guidelines publication. Three studies published between 2010 and 2014 showed no significant difference in survival or recurrence rate in patients with WDTC > 1 cm after lobectomy relatively to TT [20, 21, 22]. ATA 2015 guidelines were influenced mainly by those studies, therefore the “winds of change” for the conservative lobectomy and completion thyroidectomy existed and might have reduced the completion rates even before the official ATA guidelines publication in the end of 2015. The observed guideline change might align with a general trend in treatment practices. Our study specifically focused on the temporal association between the guideline update and the reduction in completion rates. The change in ATA guidelines might not be the sole factor contributing to the observed reduction. The study design cannot claim causality, but rather highlight this correlation.

We did not restrict our analysis to countries exclusively following the ATA guidelines, although including studies from diverse regions may introduced heterogeneity. A high completion rate was noted in India. There are several potential reasons that might better explain this discrepancy in completion rates, such as healthcare infrastructure, patient compliance, and cultural factors. The ATA 2015 guidelines affect the national guidelines in many countries outside the USA. Some countries closely follow the ATA 2015 guidelines while others adapted ATA thyroid cancer guidelines to local contexts. The influence of the ATA 2015 guidelines is evident by the absence of specific guidelines from the European Thyroid Association. However, countries such as the UK, Italy, German, Korea and India have published national guidelines regarding WDTC. UK's NICE guidelines, suggest total thyroidectomy for a T3 or T4 stage primary tumours, regional lymph node involvement, adverse pathological features, or in distant metastatic disease [23]. The Italian consensus on diagnosis and treatment of differentiated thyroid cancer: joint statements of six Italian societies suggest performing a completion thyroidectomy whenever a total thyroidectomy would be suggested as the initial surgical treatment, contralateral nodules are present, tumour size is greater than 4 cm, presence of extrathyroidal extension, lymph node metastases, or aggressive variants [24]. The German Association of Endocrine Surgeons practice guidelines for the surgical management of malignant thyroid tumours recommend total thyroidectomy in cases of lymph nodes involvement, tumours larger than 1 cm, and aggressive variants [25].

The Indian Thyroid Cancer Guidelines Task Force recommends total thyroidectomy for contralateral tumours, aggressive variants or bad pathological features, extrathyroidal extension, or extensive nodal disease [26].

Despite established guidelines, there is significant variation in the management of thyroid cancer across different countries and even within individual countries. Obviously, individual clinicians' practices may vary even within the same country. Factors contributing to this variability include differing practice patterns, concerns about disease recurrence, and patients' habits. Some recommendations from the 2015 ATA guidelines are weak due to low‐quality evidence, making uniform implementation challenging. Fear of cancer recurrence influences decision‐making. For instance, some practitioners continue to use radioactive iodine (RAI) for low‐risk micropapillary carcinoma, despite evidence against its routine use. High‐quality clinical trials are essential to address areas of uncertainty in thyroid cancer management and international collaboration and patient participation are crucial for generating robust evidence. In summary, while guidelines exist, achieving uniform implementation remains a challenge. Efforts to address barriers, improve patient education, and conduct high‐quality research are essential for optimising thyroid cancer care worldwide [27].

Our study is the first systematic review and meta‐analysis which focuses specifically on the early completion total thyroidectomy surgery rates since the ATA 2015 guidelines. Vargas‐Pinto et al. published a review in 2019 presenting studies conducted after the ATA 2015 guidelines with recommendations about the number of lobectomy patients that should complete their total thyroid resection [28]. However, the study included articles in which the surgeries were performed before 2015 and the decision about completion thyroidectomy was made theoretically, post‐factum in adherence with the new ATA 2015 guidelines. Our study tried to examine *de facto*, the trends in completion thyroidectomy since January 2016.

Another part of the review consisted of articles describing lobectomy and completion thyroidectomy surgeries among patients with a malignant and benign thyroid tumour on final pathology in the same study. A high degree of heterogeneity was observed between the completion thyroidectomy rates among the different studies. Importantly, the percentage of proven thyroid malignancy spanned from 4.6% to 97% among 20 studies [29, 30]. Consequently, the completion thyroidectomy rates were vastly diverse between studies, as the decision about the extent of the surgery is influenced by the malignant or benign pathology of the tumour. For example, Rago et al. from 2014 reported a malignancy rate of 24% and a completion thyroidectomy rate of 10.6%, while Linhares et al. from 2020 includes 82% malignant tumour patients and a completion rate of 9.5% [31, 32]. In addition, we found only one study which included surgeries performed after the 2015 ATA guidelines [32]. Due to these reasons we could not draw up clear and significant conclusions regarding the changes in the completion thyroidectomy rate among these mixed studies of malignant and benign pathologies.

### 4.1 Limitations

The majority of studies presented in this review were retrospective; some were conducted after the ATA 2015 guidelines publication, but included surgeries performed before the year 2015. Unfortunately, only a handful of studies on completion thyroidectomy surgeries performed exclusively after the new ATA guidelines were published. Eventually, we presented only four articles with malignant only completion thyroidectomy surgeries performed after 2016, and another four studies including surgeries performed during a time period of the ATA 2015 guidelines.

Our systematic review aimed to collect and analyze completion rate data. However, a more suitable study design might involve examining a large database to compare thyroidectomy completion rates before and after the release of the ATA 2015 guidelines. Alternatively, a retrospective study could explore changes in data from a single institution or, ideally, multiple institutions.

### 4.2 Study Strengths

This is the first systematic review examining real‐world data after the publication of the ATA 2015 guidelines. We implemented rigorous inclusions and exclusion criteria for studies that included malignant only lesions and separately studies that included malignant and benign tumours.

## 5 Conclusions

Our systematic review and meta‐analysis found that the rate of early completion thyroidectomy surgery was reduced by 17% since the ATA published the 2015 guidelines concerning the management of adult patients with thyroid nodules and well‐differentiated thyroid cancer, relative to the period before the recommendation publication. It seems that more surgery centres worldwide incorporated and implemented the guidelines and chose a more conservative approach concerning completion of a lobectomy to total thyroidectomy.

## Author Contributions

**Daniel Soibelman:** data curation, formal analysis, methodology, validation, visualisation, writing – original draft. **Ohad Ronen:** conceptualization, data curation, formal analysis, investigation, methodology, project administration, supervision, validation, visualisation, writing – original draft and review and editing.

## Ethics Statement

The authors have nothing to report.

## Conflicts of Interest

The authors declare no conflicts of interest.

## Data Availability

Data sharing is not applicable to this article as no new data were created or analyzed in this study.
